# Biological functions and therapeutic potential of SRY related high mobility group box 5 in human cancer

**DOI:** 10.3389/fonc.2024.1332148

**Published:** 2024-05-21

**Authors:** Juan-di Xue, Wan-fang Xiang, Ming-qin Cai, Xiao-yun Lv

**Affiliations:** ^1^ The School of Basic Medicine Sciences of Lanzhou University, Lanzhou, China; ^2^ School/Hospital of Stomatology of Lanzhou University, Lanzhou, China

**Keywords:** SRY associated high mobility population box 5 (SOX5), oncogene, cancer, biomarker, targeted therapy

## Abstract

Cancer is a heavy human burden worldwide, with high morbidity and mortality. Identification of novel cancer diagnostic and prognostic biomarkers is important for developing cancer treatment strategies and reducing mortality. Transcription factors, including SRY associated high mobility group box (SOX) proteins, are thought to be involved in the regulation of specific biological processes. There is growing evidence that SOX transcription factors play an important role in cancer progression, including tumorigenesis, changes in the tumor microenvironment, and metastasis. SOX5 is a member of SOX Group D of Sox family. SOX5 is expressed in various tissues of human body and participates in various physiological and pathological processes and various cellular processes. However, the abnormal expression of SOX5 is associated with cancer of various systems, and the abnormal expression of SOX5 acts as a tumor promoter to promote cancer cell viability, proliferation, invasion, migration and EMT through multiple mechanisms. In addition, the expression pattern of SOX5 is closely related to cancer type, stage and adverse clinical outcome. Therefore, SOX5 is considered as a potential biomarker for cancer diagnosis and prognosis. In this review, the expression of SOX5 in various human cancers, the mechanism of action and potential clinical significance of SOX5 in tumor, and the therapeutic significance of Sox5 targeting in cancer were reviewed. In order to provide a new theoretical basis for cancer clinical molecular diagnosis, molecular targeted therapy and scientific research.

## 1 Introduction

Cancer is the greatest threat to public health and one of the highest mortality rates in the world (1). Although the world economy and medical level have been continuously optimized, the global incidence and mortality of cancer have increased year by year (2). According to the annual report of the World Health Organization (WHO), it is expected that in 2030, there will be 19.3 million new cancer cases and about 10 million cancer deaths worldwide (3). Cancer is a genetic disease caused by epigenetic/genetic changes in the body’s cells (4). The occurrence of cancer is due to the changes of oncogenes (proto-oncogenes and tumor suppressor genes) caused by exogenous and endogenous factors, resulting in gene mutation or deletion, numerical or structural chromosomal alterations or epigenetic changes (5–7). At present, traditional techniques such as chemotherapy, radiotherapy and surgery are mainly used in cancer treatment (8). Although these methods have achieved relatively significant results in a short period of time, the recurrence rate and mortality of cancer are still high due to the characteristics of cancer metastasis and recurrence (9–11). In order to improve the survival rate, cure rate and mortality rate of cancer patients, molecular targeted therapy and gene therapy have become popular treatment methods for cancer (12). In order to improve the therapeutic effect and patient prognosis, it is necessary to focus on understanding the mechanism of cancer formation and development, and take this information as a starting point to find new effective biomarkers and therapeutic methods to accurately predict the prognosis of patients and carry out precision treatment (13), and constantly develop new targets to develop new drugs and combination treatment strategies (14). This is of great significance for the prevention, treatment and prognosis of cancer.

**Figure 1:**
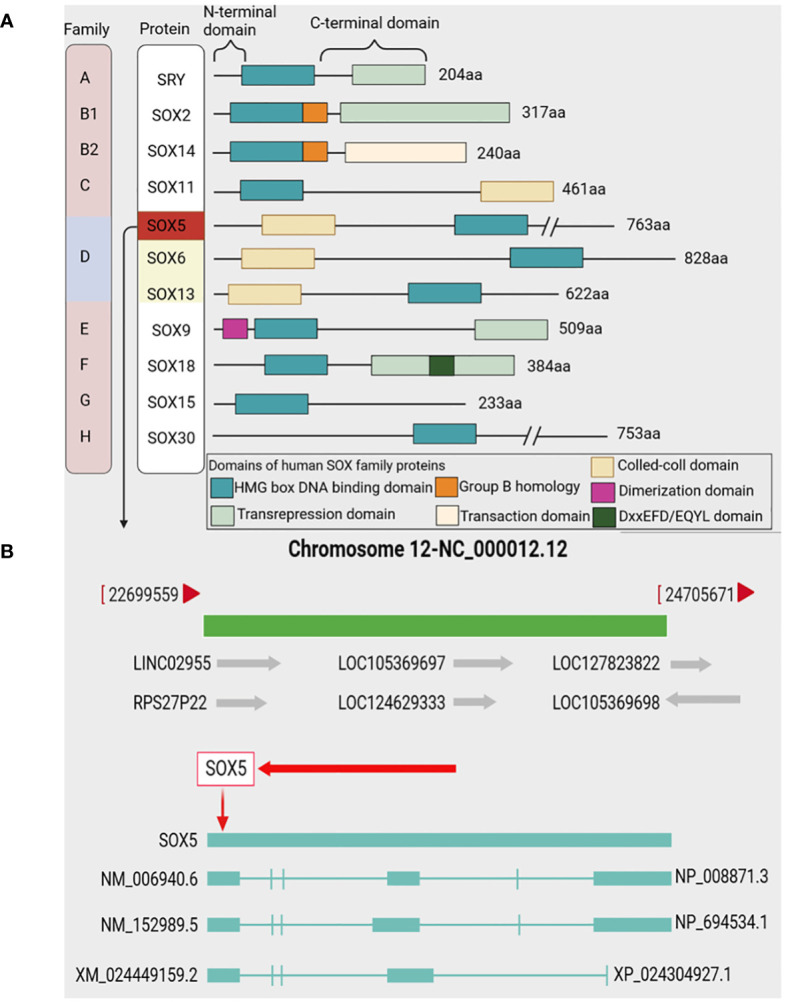
SOX family and SOX5 gene. **(A)** Representative protein structures of SOX family members from groups A to H Top from left to right: SOX family, representative members, n-terminal, C-terminal. In the bottom box, each subfamily represents the individual domains of the protein. **(B)** The SOX5 gene is located on chromosome 12q12.12 and contains 3 transcripts: NM_006940.6, NM_152989.5 and XM_0024449159.2.

The High mobility family box (SOX) transcription factor family associated with the Y chromosome of vertebrates, consisting of more than 20 members, is divided into eight groups, represented as SOXA to SOXH (15, 16) (**Figure 1**). SOX genes are defined as genes that contain evolutionarily conserved high mobility group (HMG) boxes (17). SRY is the only member of the SOXA family that plays an important role in sex determination (18). The SOX B group is divided into SOX B1 and SOX B2 subgroups, SOX B1 factors (including SOX1, SOX2 and SOX3), and SOX B2 factors include SOX14 and SOX21 (16, 19). SOX C group consists of SOX4, SOX11 and SOX12 members (20). Members of the SOX D group include SOX5, SOX6 and SOX13 (21). SOX E group includes SOX8, SOX9 and SOX10, which are proteins essential for cartilage formation (22). SOX F proteins include SOX7, SOX17 and SOX18, which play an important role in angiogenesis, cardiogenesis and lymphangiogenesis (23). SOX15 (also known as SOX20) is the only member of the SOX G group (24). SOX H group forms a new set of SOX transcription factor proteins (25). Among them, SOX2, SOX4, SOX5 and SOX9 are important members of the SOX family and are also the transcription factors that have been extensively studied at present (26). SOX transcription factor proteins play a critical role in development, binding to pre-bent DNA in nucleosomes and involved in regulating a variety of different cellular events, including the development of the retina, central nervous system, and cardiovascular system, chondrocyte differentiation, and primary sex determination (27–29). In addition, SOX is also associated with a variety of cancers, and its abnormal expression (up-regulated or down-regulated) may lead to cancer progression (30). SOX transcription factor proteins controls cell differentiation, organogenesis and many other developmental processes by encoding transcription factors with DNA-binding domains, affecting the occurrence and development of tumors (31, 32).

The SRY-related high mobility family Box 5 (SOX5) is a member of the Sox family and belongs to SOXD group (33). SOX5 is located on chromosome 12p12.12, and there are two subtypes of long SOX5 (L-SOX5) and short SOX5 (S-SOX5) (21, 34) (**Figure 1**). Previous studies have shown that SOX5 is expressed in a variety of human tissues, including cartilage, brain, heart, liver, and muscle (33). SOX5 is involved in various physiological and pathological processes and a variety of cellular processes, including the regulation of embryonic development, chondrogenesis, neocortical development and differentiation of immune cells, as well as cell growth, apoptosis and cell cycle (35–37). The TCGA database shows that the abnormal expression of SOX5 acts as an oncogene to promote the occurrence and development of tumors and the maintenance of cancer cell phenotypes. Meanwhile, many studies have confirmed that SOX5 plays an important role in the progression of various cancers (38, 39). Abnormal expression of SOX5 in human cancer is closely related to the occurrence, development, migration, metastasis and prognosis of cancer (40, 41), and SOX5 knockout can induce cancer cell cycle arrest and apoptosis, and inhibit the progression and migration of cancer (42). This suggests that cancer-associated SOX5 can be used not only as a reliable diagnostic marker, but also as a potential therapeutic target. In this review, we summarize the role, mechanism and potential clinical significance of SOX5 in cancer pathology. It provides a new theoretical basis for clinical molecular diagnosis, molecular targeted therapy and scientific research of cancer.

## 2 SOX5 expression in cancer

**Figure 2:**
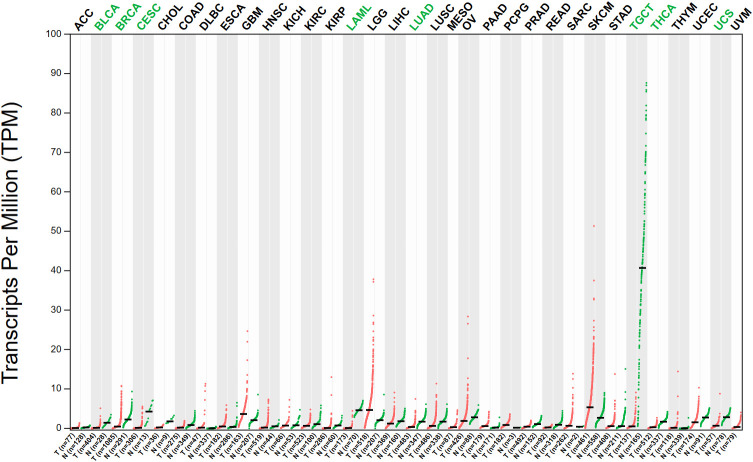
The expression of SOX5 in various tumors was different from that in normal tissues. (Data from GEPIA (Gene Expression Profiling Interactive Analysis). N, Normal tissue; T, tumor tissue; ACC, adrenocortical cancer; BLCA, bladder urothelial carcinoma; BRCA, breast invasive carcinoma; CESC, cervical & endocervical cancer; CHOL, cholangiocarcinoma; COAD, colon adenocarcinoma; DLBC, diffuse large B-cell lymphoma; ESCA, esophageal carcinoma; GBM, glioblastoma multiforme; HNSC, head & neck squamous cell carcinoma; KICH, kidney chromophobe; KIRC, kidney clear cell carcinoma; KIRP, kidney papillary cell carcinoma; LAML, acute myeloid leukemia; LGG, brain lower grade glioma; LIHC, liver hepatocellular carcinoma; LUAD, lung adenocarcinoma; LUSC, lung squamous cell carcinoma; MESO, mesothelioma; OV, ovarian serous cystadenocarcinoma; PAAD, pancreatic adenocarcinoma; PCPG, pheochromocytoma & paraganglioma; PRAD, prostate adenocarcinoma; READ, rectum adenocarcinoma; SARC, sarcoma; SKCM, skin cutaneous melanoma; STAD, stomach adenocarcinoma; TGCT, testicular germ cell tumor; THCA, thyroid carcinoma; THYM, thymoma; UCEC, uterine corpus endometrioid carcinoma; UCS, uterine carcinosarcoma; UVM, uveal melanoma. The abbreviation written in green indicates that the expression of SOX5 is higher in normal tissue of this tumor than in cancerous tissue.

The expression pattern of SOX5 has been studied in a wide range of cancers and has been shown in the TCGA database to be significantly altered in a variety of cancers compared to normal tissues (**Figure 2**). As a transcription factor, it binds to specific DNA sequences and activates gene transcription (43). It has a key function in regulating embryonic development and determining cell fate (44). Existing experiments have shown that SOX5 is highly expressed in many malignant tumors, including prostate cancer, breast cancer, hepatocellular carcinoma, nasopharyngeal carcinoma and other malignant tumors (45–47). Evidence from TCGA database shows that Sox5 expression is up-regulated in prostate, and patients with high SOX5 level are more likely to develop metastasis and have lower survival rate (39). In breast cancer, SOX5 is highly expressed in breast cancer tissues compared with adjacent healthy tissues, and overexpression of SOX5 is associated with decreased overall survival of breast cancer patients (48). In hepatocellular carcinoma, SOX5 is up-regulated in hepatocellular carcinoma tissues and cell lines, and high levels of SOX5 can accelerate the migration and invasion of HCC cells *in vitro* (45). In nasopharyngeal carcinoma, SOX5 is significantly up-regulated in cancer cells, and the high expression of SOX5 promotes the proliferation and migration ability of nasopharyngeal carcinoma cells, and is negatively correlated with the survival rate of patients with nasopharyngeal carcinoma (47). In addition, SOX5 also plays an abnormally high expression role as an oncogene in many other cancers (such as gastric cancer, lung cancer, ovarian cancer and colorectal cancer, etc.), thus regulating the occurrence, development and pathological process of cancer (49, 50). Meanwhile, abnormal expression of SOX5 can also promote cancer proliferation, invasion and Epithelial to Mesenchymal Transition (EMT) by targeting different downstream genes such as Twist1, Snail and acidic secreted protein rich in cysteine (51–53). This suggests that the abnormal expression of SOX5 plays an important role in the process of carcinogenesis.

## 3 SOX5 in multiple human cancers

More and more evidence shows that SOX5 is abnormally expressed as an oncogene in cancers of multiple human systems and involved in the occurrence and development of cancer (**Table 1**). This chapter mainly introduces the expression of SOX5 in related cancers and its influence on cancer.

**Table 1:** The role of S SOX5 in cancer and its related mechanisms.

Tumors	Expression of Sox5	Findings of SOX5	Effect of high expression in cancer	Inhibition of Sox5 in cancer	Reference
Gastric cancer	High expression	*In vitro* and in patients	Promote proliferation, invasion and drug resistance and low survival rate	Inhibit proliferation, invasion and promote apoptosis and improve patient survival rate	(54, 55)
Liver cancer	High expression	*In vitro* and in patients	Promote proliferation, invasion and drug resistance	Inhibit the proliferation and arrest cell cycle and improve patient survival rate	(45, 56)
Bladder cancer	High expression	*In vitro* and in patients	Promote the migration, invasion and drug resistance and low survival rate	Inhibit the migration, invasion and promote apoptosis and improve patient survival rate	(40)
Prostatic cancer	High expression	*In vitro* and in patients	Promote cancer cell proliferation, migration and invasion	Inhibits proliferation, migration, invasion and improve patient survival rate	(57, 58)
Breast Cancer	High expression	*In vitro* and in patients	Promote proliferation, migration and low survival rate	Inhibit cancer cell proliferation, migration and improve patient survival rate	(59, 60)
Lung cancer	High expression	*In vitro* and in patients	Promote migration, invasion and promote the development of lung cancer	Inhibits migration, invasion and promotes apoptosis	(61, 62)
Nasopharynx cancer	High expression	*In vitro* and in patients	Promote proliferation, migration and invasion	Delay cell cycle and inhibit cell apoptosis	(47)
Glioma	High expression	*In vitro* and in patients	Promote the migration and invasion of cancer cells	Inhibit migration and invasion	(63)
Osteosar-coma	High expression	*In vitro*	Promote proliferation, invasion and low survival rate	Inhibits cancer cell migration and invasion	(42)

### 3.1 Hepatocellular carcinoma

Hepatocellular carcinoma (HCC) is a common malignancy of the digestive system and the third leading cause of cancer-related death in the world, with nearly 1 million new cases of HCC worldwide every year (64). HCC is often accompanied by lymphatic and/or blood metastasis and invasion of surrounding tissues and organs, which seriously threatens human life and health (65, 66). Although the diagnosis and treatment of HCC has progressed in recent years, its overall survival rate remains low. Therefore, it is urgent to find new biomarkers and therapeutic targets for early diagnosis (67). The role of Twist1 in promoting aggressiveness and EMT processes has been widely reported in cancer. In HCC, the expression of SOX5 is significantly increased in HCC tissues and cell lines, and its high expression induces EMT by up-regulating Twist1 expression, thereby inducing invasion and metastasis of HCC (56). Wang et al. found that SOX5 expression is significantly upregulated in HCC tissues and cell lines, and overexpressed SOX5 in HCC cancer cells significantly promotes cell migration and invasion. After silencing SOX5, the mRNA and protein expressions of mesenchymal phenotypic markers N-cadherin, vimentin and fibronectin can be significantly down-regulated, and the epithelial phenotypic marker E-cadherin can be up-regulated, thereby inhibiting EMT. In addition, silencing SOX5 also causes cancer cell spindles to deform less and pseudopods to be shorter (45).

### 3.2 Gastric cancer

Gastric cancer (GC) is one of the most common malignancies in the world and the third leading cause of cancer-related death (68). Despite continuous advances in the diagnosis and treatment of GC, the 5-year survival rate of most GC patients is less than 30% due to the lack of specific biomarkers for early GC diagnosis (69). Zheng et al. found that in GC, compared with normal tissues, SOX5 mRNA expression increased in tumor tissues and *in vitro* cells of GC patients, and high expression of SOX5 significantly promoted the proliferation and migration of cancer cells (54). In addition, You et al. also found that upregulation of SOX5 expression in GC specimens was significantly associated with clinical metastasis and poor prognosis in GC patients, and SOX5 promoted GC cell invasion and metastasis through activation of Twist-mediated EMT (55). These studies indicate that SOX5 plays an important role in GC, and inhibiting its expression is conducive to inhibiting the occurrence and development of GC.

### 3.3 Bladder cancer

Bladder cancer (BC) is one of the most common and life-threatening cancers in the global male population, with high morbidity and mortality (70, 71). Despite significant advances in the diagnosis and treatment of BC, the lack of specific diagnostic and therapeutic markers has led to a high mortality rate in BC patients (72). Wu et al. found that the expression of SOX5 increased in BC tissues and *in vitro* cell lines, and high expression of SOX5 could promote the growth and migration of BC cancer cells, while inhibition of SOX5 would inhibit the expression of DNA methyltransferase 1 (DNMT1), thus inhibiting the growth and migration of cancer cells and promoting cell apoptosis (40).

### 3.4 Prostate cancer

Prostate cancer (PCa) is a common malignant tumor and one of the malignant tumors affecting men (73, 74). Genomics studies have identified certain molecules as important oncogenes that contribute to the occurrence and development of PCa, such as SOX5 (75). Yang et al. found that SOX5 mRNA was elevated in tumor tissues of prostate cancer patients compared with normal tissues. High expression of SOX5 promotes cell proliferation and migration of PCa cancer cells (57). Moreover, in prostate cancer, evidence from the TCGA database suggests that SOX5 is associated with prostate progression. Hu et al. found that SOX5 is highly expressed in PCa, and high levels of SOX5 can promote PCa cell metastasis, and can also induce PCa EMT to promote tumor metastasis by regulating the expression of Twist1. Meanwhile, high levels of SOX5 were negatively correlated with PCa patient-specific survival (58).

### 3.5 Breast cancer

Breast cancer (BC) is one of the most common malignant tumors in women worldwide, and its incidence has been increasing in recent years, with about 1.7 million newly diagnosed cases of breast cancer worldwide every year (76, 77). Although the mortality rate of breast cancer is decreasing year by year, breast cancer is prone to invasion and metastasis, which leads to treatment failure for breast cancer patients (78). Pei et al. found that SOX5 may be a potential oncogene, which is highly expressed in breast cancer cells, and high expression of SOX5 can promote the proliferation, migration and invasion of cancer cells, while knockout of SOX5 can inhibit the EMT program of human breast cancer cells, and thus inhibit the proliferation, migration and invasion of cancer cells (59). In addition, a recent study also found that Sox5 expression is associated with bone metastasis of breast cancer. Chen et al. used chromatin immunoprecipitation (ChIP) detection to find that SOX5 is the direct downstream target gene of Brachyury, and Brachyury directly regulates SOX5 expression by binding to two moieties in the SOX5 promoter region. However, knocking down SOX5 can reverse the colonization and survival ability of cancer cells in bone matrix (60). In addition, other studies have found that the enhancer of zeste 2 multiplex inhibitory complex subunit 2 (EZH2) acts as the downstream target gene of SOX5, and the ectopic expression of SOX5 increases the expression of EZH2 at the mRNA and protein levels, while the knockdown of SOX5 can reduce the expression of EZH2. Thus inhibiting the proliferation and invasion of breast cancer (48).

### 3.6 Lung cancer

Lung cancer is one of the most common malignant tumors in the world and the leading cause of cancer-related death worldwide (79, 80). Chen et al. used immunohistochemical analysis to find that SOX5 is overexpressed in lung adenocarcinoma, and overexpressed SOX5 can accelerate the progression and metastasis of lung adenocarcinoma through EMT, and is also correlated with clinical stage, poor prognosis and overall survival time of LAC patients. However, knocking out SOX5 can inhibit the proliferation and metastasis of lung cancer cells (51). Non-small cell lung cancer (NSCLC) is the most common histological type of lung cancer (81). Many studies have suggested that SOX5, as an oncogene, is involved in the occurrence and development of NSCLC (61). Li et al. found that SOX5 was highly expressed in tissues and cell lines of NSCLC, and highly expressed SOX5 could promote the development of NSCLC (62), and suggested that the mechanism might be to drive the malignant potential of NSCLC through interaction with YAP1 (82).

### 3.7 Glioma

Glioma is the most common tumor of the central nervous system, accounting for more than 60% of primary brain tumors (83). It has been reported that members of Sox gene family group E and Group D are important transcriptional regulators of glial development in the central nervous system (84). UEDA R et al. found that SOX5 was abnormally high expressed in glioma, but only a few SOX5-positive cells were detected in non-tumor tissues of the cerebral cortex. In addition, high expression of SOX5 was negatively correlated with the survival rate of glioma patients (63).

### 3.8 Osteosarcoma

Osteosarcoma is the most common primary bone tumor in children and adolescents, seriously affecting their survival (85). Although the combination of surgery and multi-target chemotherapy has greatly improved the overall survival rate of patients with osteosarcoma, chemotherapy resistance remains a barrier to treatment (86). Therefore, there is an urgent need to find new therapeutic targets for osteosarcoma. SOX5 has been shown to be highly expressed in osteosarcoma and act as a tumor promoter (87). Snail overexpression in cancer cells affects cell survival, angiogenesis, and chemotherapy resistance *in vitro*, and promotes *in vivo* metastasis (88). Zhang et al. found that Sox5 expression was significantly upregulated in osteosarcoma tissues and cell lines (MG63 and U2OS) and was associated with cell migration and invasion. Abnormally expressed SOX5 promotes EMT by up-regulating Snail, and thus significantly promotes osteosarcoma cell migration (p < 0.05) and invasion (p < 0.05) (42).

### 3.9 Nasopharyngeal carcinoma

Nasopharyngeal carcinoma (NPC) is a serious malignant tumor originating from the nasopharyngeal epithelium, and the prevalence of NPC is high in East and Southeast Asia (89). Previous studies have found that SOX5, as an oncogenic gene, is involved in the development of various cancers. Huang et al. found that SOX5 is significantly up-regulated in nasopharyngeal carcinoma tissues and cells, and the overexpression of SOX5 can promote the proliferation and migration of NPC cells. Overexpression of SOX5 in tumor cells is also clinically associated with poor survival of patients (47). The above studies show that SOX5 plays a role in promoting the biological processes of proliferation, migration and invasion of various cancer cells, which may provide a potential new tool for the clinical diagnosis and treatment of cancer in the future.

## 4 Biological role of SOX5 in human cancer

**Figure 3:**
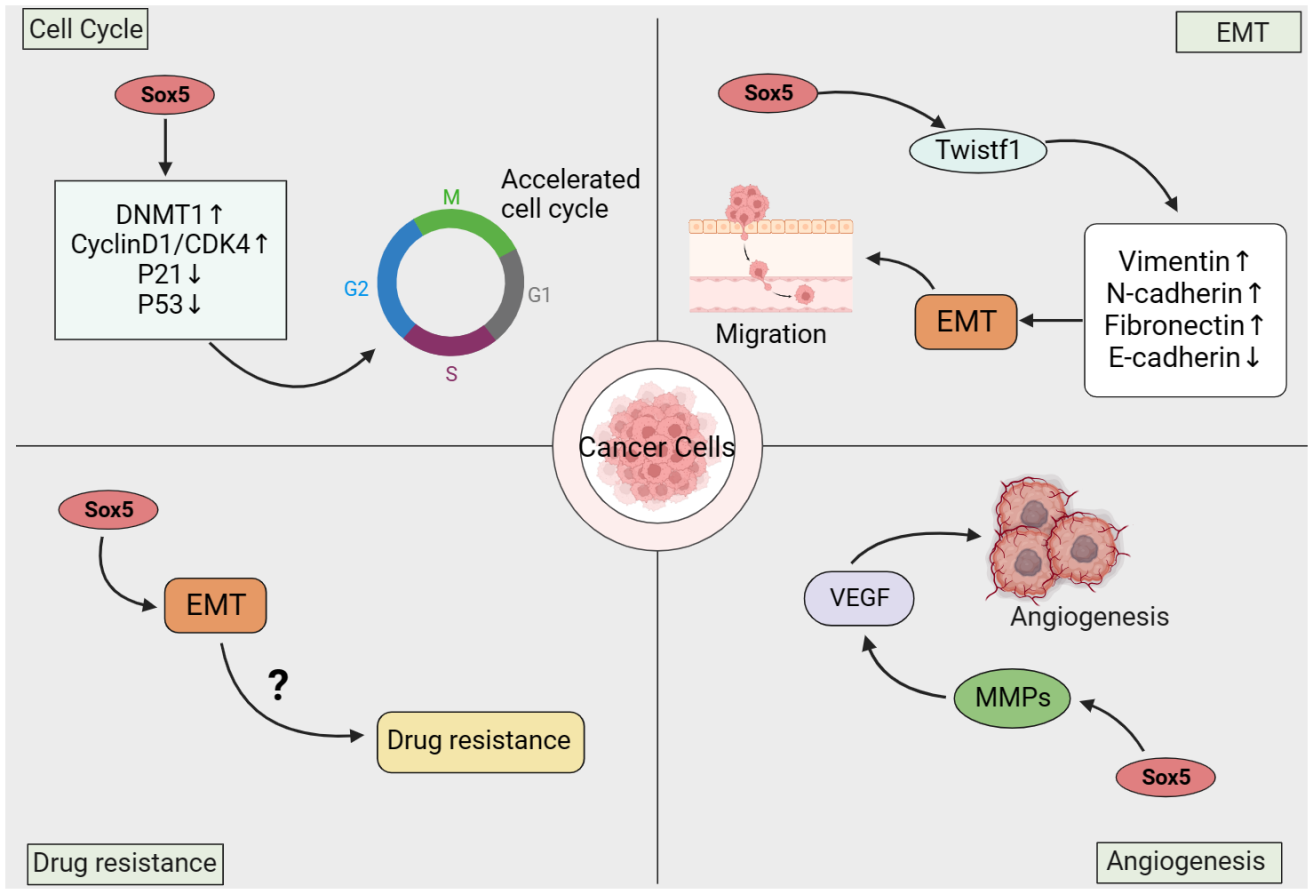
Mechanism of action of SOX5 in cancer.

The occurrence and development of cancer is a complex process, including excessive proliferation of cancer cells, anti-apoptosis, invasion and metastasis, and neovascularization (90). As an oncogene, the abnormal expression of SOX5 not only regulates the occurrence and development of various cancers, but also mediates the proliferation, metastasis, epithelial-mesenchymal transformation (EMT) and angiogenesis of cancer cells (91) (**Figure 3**).

### 4.1 SOX5 and cell cycle and proliferation

Cell cycle and proliferation are closely related to the aggressive biological behavior of malignant tumors and are basic markers of cancer progression (92). Studies have reported that SOX5 is involved in a variety of cellular processes, including cell growth, apoptosis, cell cycle, cell senescence, senescence and metabolism (27). Cyclin-dependent inhibitor p21, as the downstream target of tumor suppressor p53, can inhibit cell growth and migration and promote cell apoptosis and cell senescence (93). In mammals, there are three types of DNA methyltransferase (DNMT) that catalyze DNA methylation (94). DNA methylation affects a variety of cellular processes, including embryonic development, cell growth and death (95). In bladder cancer, SOX5 expression is up-regulated in bladder cancer tissues and *in vitro* cell lines. Highly expressed SOX5 can promote the expression of DNMT1 and inhibit the expression of p21 through DNA methylation, thus promoting cell growth and migration, while knockdown of Sox5 can inhibit the cell growth and migration of bladder cancer cells (40). SOX5 can promote cyclin D1/cyclin dependent kinase 4 (CDK4) complex, cyclin A and Rb phosphorylation, thereby reducing the percentage of G0/G1 phase cells and increasing the percentage of S phase cells, thereby promoting cancer cell proliferation (48).

### 4.2 Migration and invasion

Cancer metastasis refers to the spread of cancer cells from the primary tumor to distant sites and is an important cause of cancer-related death (96). Epithelial to Mesenchymal Transition (EMT) plays a key role in cancer invasion potential and can promote cancer metastasis (97). EMT means that polarized fixed epithelial cells transform into fusiform mobile mesenchymal cells with cell migration and invasion capabilities (98). Previous studies have confirmed that the abnormal expression of SOX5 can promote the epithelial-stromal transformation (EMT), proliferation and invasion of cancers, including hepatocellular carcinoma, breast cancer, stomach cancer and prostate cancer, by targeting different downstream genes (such as Twist1, Snail and ZEBI) (55, 57). As an oncogene, SOX5 significantly upregulates the expression of EMT-related genes, such as Twist and ZEBI, thereby promoting EMT (59). Twist is the upstream mediator of EMT, which can induce cancer metastasis (99). Increased Twist in cancer cells has been shown to promote cell survival, angiogenesis, and chemotherapy resistance *in vivo* and *in vitro* (100). Twist1 is a member of the basic helix ring helix transcription factor and an important transcription factor of EMT. It is overexpressed in various solid tumors in humans, including sarcoma, glioma, neuroblastoma, and melanoma (101, 102). As a transcription factor, SOX5 regulates the expression of Twist1 by binding to conserved Sox5 binding sites in the Twist1 promoter, and Twist1 promotes tumor metastasis by inducing EMT (59, 103). Studies have also found that exogenous overexpression of Twist1 increases the invasion and metastasis of cancer cells by promoting the down-regulation of E-cadherin and induction of EMT (104, 105). For example, in osteosarcoma, SOX5 overexpression promotes EMT by regulating Snail (42), and can increase the level of EMT markers in gastric and lung cancer to enhance cell migration and invasion (51, 55). In addition, studies have shown that the transforming growth factor-β (TGF-β) signaling pathway is an effective inducer of EMT and plays a key role in cancer metastasis (106). TGF-β can induce SOX5 expression through Smad3 phosphorylation, and SOX5 can promote the expression of Twist1, resulting in overexpression of Twist1 and inducing EMT to promote tumor metastasis, while targeting SOX5 can effectively delay TGF-β signal-induced EMT (58). Further studies have shown that SOX5 can directly or indirectly down-regulate the expression of E-cadherin, up-regulate the protein expression of N-cadherin, Vimentin and fibronectin, thus promoting the occurrence of cancer EMT. Knocking down SOX5 can inhibit EMT by up-regulating e-cadherin and down-regulating n-cadherin, vimentin, and fibronectin (59).

### 4.3 Drug resistance

Tumor cells often have or develop resistance to cytotoxic drugs, which explains the lower success rates of drug therapies for some types of cancer. A series of studies have identified the important role of SOX5 in the development of resistance to chemotherapy, radiotherapy and targeted therapy in different types of cancer cells (107). Cisplatin is the main systemic chemotherapy treatment for some cancer patients (108). Studies have found that the high expression of SOX5 in gastric cancer can increase the resistance of cancer cells to cisplatin, while inhibiting the expression of SOX5 can alleviate or reverse the resistance to cisplatin (109). In addition, SOX5 has been discussed as a resistance factor in docetaxel-resistant lung adenocarcinoma cells, where SOX5 plays a key role in exacerbating the development of EMT, metastasis, and chemotherapy resistance in docetaxel-resistant lung adenocarcinoma cells. Inhibition of SOX5 can inhibit cell proliferation, migration and EMT process to reverse chemotherapy resistance of docetaxel-resistant lung adenocarcinoma cells (110).

### 4.4 Angiogenesis

Angiogenesis (development of new blood vessels) is a key process of tumor cell growth and progression, and is crucial in the process of cancer cell proliferation and metastasis, which is the main cause of poor prognosis (111). A large number of studies have shown that SOX gene plays a crucial role in angiogenesis. For example, SOX5, SOX7, SOX9 and SOX17 (112–114). VEGF induces tumor angiogenesis as a pro-angiogenic factor, which is closely related to the occurrence, development and metastasis of cancer (115). Vascular endothelial growth factor (VEGF) is an important protein in new blood vessel formation (116), and Sox5 may play an important role in VEGF-induced angiogenesis (117). Chen et al. found that SOX5 can promote angiogenesis by regulating VEGF expression through *in vitro* Sox5 knockdown and overexpression analysis and tube formation experiment. In addition, through the analysis of 90 clinical lung adenocarcinoma samples, it was found that VEGF expression was positively correlated with SOX5 expression, and the overexpression of SOX5 led to the activation of STAT3, which would increase the expression of VEGF and stimulate angiogenesis (118).

## 5 Clinical significance of SOX5 in human tumors

### 5.1 SOX5 as biomarker for cancer diagnosis and prognosis

Biomarkers are of great significance for the diagnosis and prognosis of cancer (67). Previous studies have highlighted the important role of the SOX family in cancer diagnosis, prognosis, and targeted therapy (119). As an oncogene, SOX5 is highly expressed in a variety of cancers. For example, SOX5 was significantly upregulated in GC tissues compared to paired adjacent non-tumor tissues. The up-regulated expression of SOX5 can promote the occurrence and development of GC, and also promote the proliferation, distant metastasis and invasion of cancer cells. At the same time, SOX5, which is highly expressed in many cancers such as prostate cancer, bladder cancer, liver cancer and breast cancer, can also promote the progression of cancer and the proliferation, migration and invasion of cancer cells. Knocking down or knocking out Sox5 in these cancers can inhibit cancer cell proliferation, migration, invasion, and tumor progression. This suggests that Sox5 may serve as a biomarker for cancer diagnosis. In addition, studies have shown that certain SOX family members whose expression is up-regulated in different forms of cancer are associated with poor prognosis (120). For example, in prostate cancer, SOX5 has been shown to be significantly upregulated in prostate cancer tissue, and overexpression of SOX5 is associated with larger tumor size, later clinical stage, and poorer prognosis (58). In gastric cancer, it was also found that the expression level of SOX5 was significantly correlated with T stage, pTNM stage and lymph node metastasis (55). Lymph node metastasis and pT and pN staging are independent prognostic factors for overall survival of patients with gastric cancer (121). In addition, high SOX5 expression has been found to be associated with poor prognosis and tumor metastasis in cancers such as lung adenocarcinoma, glioma, and nasopharyngeal carcinoma. These studies suggest that SOX5 may serve as a prognostic marker for cancer. Although SOX5 has not yet been reported to be specific and sensitive in which type of cancer than currently used diagnostic or prognostic markers, it is not difficult to find that SOX5 appears to be a promising diagnostic and prognostic factor.

### 5.2 SOX5 is a promising target for cancer therapy

In recent years, gene therapy has made great progress in improving the treatment of various genetic diseases and cancers due to its flexibility, high efficiency and reduction of off-target effects (122). Indeed, the same biochemical characteristics that make SOX5 a suitable biomarker may make it a promising therapeutic target. As previously mentioned, the biological function and clinical significance of SOX5 in cancer provide an opportunity for SOX5 to be a promising target for cancer therapy. In PCa, SOX5 is significantly increased in primary PCa tissues compared with normal tissues, and plays an important role in PCa invasion and metastasis, which can be significantly inhibited after SOX5 knockout (58). Studies have shown that SOX5 is described as a potential therapeutic target for HCC metastasis in HCC, and high expression of SOX5 can promote HCC cell migration and invasion (45). SOX5 is significantly upregulated in primary lung cancer tissues and acts as a tumor metastasis promoting gene in lung cancer, and its overexpression promotes cell invasion and metastasis, while inhibiting SOX5 can inhibit tumor metastasis promoting genes in lung cancer (51). In gastric cancer, You et al. also found that SOX5 expression in GC specimens was up-regulated with clinical metastasis in GC patients and promoted GC cell invasion and metastasis through activation of Twist-mediated EMT (55). These studies suggest that SOX5 may be a potential target for treatment, and that controlling SOX5 levels could be a promising cancer treatment.

## 6 Regulation of SOX5 by non-coding RNAs

Non-coding RNAs (NcRNAs) plays an indispensable role in the growth and development of organisms through its influence on transcription and translation (123). The clinical application of ncRNAs as potential therapeutic targets for cancer can be manifested in two situations: the use of Ncrnas to “replenish” suppressed or missing RNA (replacement therapy) or to “block” the action of overactive cancer-causing RNA (124). Some studies believe that SOX5 is the target gene of multiple ncRNAs, and a variety of ncRNAs can target SOX5 to play a therapeutic role in cancer (125). MicroRNAs (miRNAs) are a class of non-coding RNAs with a length of about 22 bp that regulate gene expression at the post-transcriptional level (126) and directly bind to the 3’ untranslated region (3’UTR) of the target messenger RNA (mRNA), thereby inhibiting translation and/or driving mRNA degradation (127, 128). Multiple miRNAs have been shown to be abnormally expressed in specific cancer types in humans, where they can exert carcinogenic or tumor suppressor effects, controlling target gene expression in a tumor type-specific manner (129). miR-338–3p acts as a tumor suppressor in gastric cancer, inhibiting cell growth, survival, and proliferation by directly targeting SOX5 and blocking Wnt/β-catenin signaling, while inducing apoptosis (54). In addition, miR-539 can inhibit the proliferation and migration of gastric cancer cells by targeting SOX5 (49). The increase of miR-139–5p greatly inhibits the expression of SOX5 in prostate cancer cells, down-regulates TWIST, and reduces the expression of N-cadherin and vimentin, thereby inhibiting the EMT process and thus inhibiting the cell proliferation and cell migration of prostate cancer cells (57). In breast cancer, miR-146a-5p is down-regulated in BC tissues and cells, while overexpression of miR-146a-5p can inhibit BC cell proliferation, migration, invasion and EMT by targeting SOX5 (125). miR-143–3p specifically inhibits SOX5 by recognizing the target sequence of SOX5 mRNA 3’-UTR in NSCLC cell lines, thereby inhibiting EMT in NSCLC (130). LncRNAs are large non-coding RNAs with >200 nucleotide transcripts and are important regulators of various disease processes (131). In HCC, the expression of LINC00520 is up-regulated in HCC tissues, promoting the proliferation, migration and invasion of HCC cells, and is negatively correlated with the survival rate of patients. LINC00520 acts as a sponge of miR-4516 to regulate SOX5, and inhibition of LINC00520 can effectively inhibit the expression of SOX5, thereby inhibiting the proliferation, migration and invasion of HCC cells (132). The competitive endogenous RNA (ceRNA) network is an important mechanism of circRNA in human disease, targeting mRNA via sponge miRNA (133). Studies have shown that circCDR1as may act as a ceRNA of miR-219a-5p to inhibit SOX5, thereby inhibiting cell viability, migration and invasion, and promoting cell apoptosis to inhibit the progression of NSCLC (62). circDOCK1 exerts its function through sponge hsa-miR-132–3p and regulates the expression of SOX5 to form the circDOCK1/hsa-miR-132–3p/SOX5 regulatory axis. Down-regulating the expression of circDOCK1 can inhibit SOX5, thereby reducing cancer cell viability, inhibiting cell proliferation and inhibiting the cell migration potential of BC cells (134) (**Table 2**). NcRNA-based alternative therapies mainly benefit patients with reduced expression of tumor suppressor miRNAs or overexpression of downstream targets of these miRNAs. For cancer patients with low expression of tumor suppressor miRNA, supplementation of down-regulated miRNAs with multiple genetic targets critical for tumorigenesis may be an attractive therapeutic modus operis. In addition, it is important to recognize that the molecular characteristics and functional roles of miRNAs vary by tumor type. Therefore, the effectiveness of miRNA therapies must be tested in individual tumor types, and the tumor-specific efficacy of these therapies needs to be elucidated.

**Table 2:** Mechanism of action of non-coding RNA targeting SOX5 in human cancer.

ncRNAs	Cancer	Expression of ncRNA	Mechanisms	Refer-ences
miR-338–3p	Gastric cancer	Low expression	miR-338–3p↑→Wnt/βcatenin↓→ Inhibit proliferation and promote apoptosis	(54)
miR-539	Gastric cancer	Low expression	miR-539↑→ Sox5 expression↓→ Inhibit proliferation and migration	(49)
miR-139–5p	Prostate cancer	Low expression	miR-139–5p↑→Twist1↓, N-cadherin↓, Vimentin↓, EMT↓→ Inhibits proliferation and migration	(57)
miR-146a-5p	Breast Cancer	Low expression	miR-146a-5p↑→Sox5 expression↓→ EMT↓→Inhibit proliferation, migration and invasion	(125)
miR-143–3p	NSCLC	High expression	miR-143–3p↑→Sox5 expression↓→ EMT↓→Inhibit proliferation, migration and invasion	(130)
LINC00520	HCC	High expression	LINC00520↓→Act as a sponge for miR-4516→Sox5 Expression↓→ Inhibit proliferation, migration and invasion	(132)
circCDR1as	NSCLC	Low expression	circCDR1as↑→Act as a sponge for miR-219a-5p→Sox5 expression↓→ Inhibit proliferation, migration and invasion and promote apoptosis	(62)
circDOCK1	Breast Cancer	High expression	circDOCK1↓→Sox5 expression↓→ Inhibit migration and invasion	(134)

## 7 Discussion

SRY related High mobility family Box 5 (SOX5) is a member of SOX Group D of the Sox family. SOX5 is widely expressed in a variety of human systems. Under normal circumstances, SOX5 is involved in various physiological and pathological processes and various cellular processes, such as cell growth, apoptosis and cell cycle. More and more evidence shows that Sox5 acts as a tumor promoter and is abnormally high expressed in a variety of human cancers. The abnormally high expression of SOX5 plays an important role in cancer progression and metastasis by promoting tumor cell viability, proliferation, invasion, migration and EMT, and it is also believed that the abnormally high expression of SOX5 is closely related to adverse clinical outcomes. However, there is no exact explanation for the specific regulatory mechanism through which SOX5 affects tumor proliferation, invasion, migration and EMT during tumor progression. Second, although Sox5 has been extensively studied in the field of cancer, current research is mainly limited to the cellular or rodent stage, and its expression level and chemical stability in body fluids have not been clearly verified. Therefore, follow-up research on SOX5 and cancer should be done: (a) Further expanding the clinical application of SOX5 as a biomarker in multicenter large sample studies requires more animal experiments and preclinical studies to further explore the specific role and clinical potential of SOX5 in cancer. (b) The mechanism by which SOX5 exerts its regulatory role in tumors, and the mechanism by which Sox5 affects the occurrence and progression of cancer.

## 8 Conclusion

In summary, the abnormally high expression of SOX5 in a variety of human malignant tumors can lead to the occurrence and development of cancer, and plays a role in almost all aspects of tumor biology (proliferation, migration, invasion, and drug resistance). Therefore, SOX5 may be a potential biomarker for cancer diagnosis and prognosis, as well as a potential target for cancer therapy.

## Author contributions

JX: Writing – original draf & review. Resources, Supervision. WX: Writing – review. MC: Data curation, Writing – review. XL: Funding acquisition, Project administration, Writing – review & editing.
